# Self-perfection or self-selection? Unraveling the relationship between job-related training and adults’ literacy skills

**DOI:** 10.1371/journal.pone.0215971

**Published:** 2019-05-01

**Authors:** Britta Gauly, Clemens M. Lechner

**Affiliations:** Department of Survey Design and Methodology, GESIS–Leibniz Institute for the Social Sciences, Mannheim, Germany; Leibniz Institute for Educational Trajectories, GERMANY

## Abstract

Can participation in job-related training contribute to the formation and maintenance of adults’ literacy skills? Although evidence suggests that participation in training is related to higher literacy skills, it remains unclear whether this association reflects a causal effect of training participation on literacy (training effects), results from the self-selection of more high-skilled individuals into training (selection effects), or is due to other sources of endogeneity (e.g., omitted variable bias). To unravel these possibilities, we used data from the Programme for the International Assessment of Adult Competencies (PIAAC) and its German follow-up, PIAAC-Longitudinal (PIAAC-L). As these unique data offer repeated measures of literacy skills, spaced three years apart, in a large and representative sample, they allowed us to disentangle training effects from selection effects and to account for potential endogeneity. Analyses revealed that, even after taking account of formal education and a host of job characteristics, individuals with higher literacy skills were more likely to participate in training. By contrast, no evidence for effects of training on literacy skills emerged in any of our models, which comprised lagged-dependent, fixed effects, and instrumental-variable models. These findings suggest that, rather than job-related training contributing to literacy development, individuals with higher literacy skills are more likely to participate in training.

## Introduction

As the old adage goes, “you can’t teach an old dog new tricks.” Or can you? This question is central to research on lifelong learning in the workplace. Here, researchers ask what occupational factors contribute to the formation or maintenance of worker’s individual skills after finishing formal education [1–3]. In the light of the growing pace of technological innovation and the demographic challenge of an ageing workforce, this question is particularly relevant both for individuals themselves (e.g., for maintaining employment prospects) and for society at large (e.g., in terms of economic growth) [4, 5].

Workplace-based learning—in particular participation in job-related training—is one of the key conduits for the development of adults’ skills [6, 7]. Job-related training is usually designed to improve job-specific or even firm-specific skills. There are, however, some indications that even highly specific training might have positive spill-over effects to an important more general skill, namely literacy. Literacy refers to “the ability to understand, evaluate, use, and engage with written texts” [8]. It is often seen as the most important foundational skill required to successfully navigate today’s increasingly cognitively complex world of work––and as an indispensable prerequisite for acquiring further human capital, including more job-specific skills [9].

The most important indications for such spill-over effects of job-related training on literacy stem from cross-sectional data that have reported positive associations between participation in training and measures of general skills, including literacy [6, 8, 10, 11]. In addition, results from a British study on participants’ perceived benefits of training participation showed that almost 90 percent of individuals participating in job-related training stated that training also helped them to improve their general skills [12]. Such spill-over effects would be consistent with practice engagement theory and related theoretical perspectives [1, 13]: To the extent that job-related training includes any type of material that requires participants to read and write, it might offer opportunities to apply and practice their literacy skills.

From a lifelong learning perspective, this would be good news. It would mean that training primarily designed to foster job-specific skills also contributes to the growth of literacy skills—or at least prevents their decline. However, as we argue here, the existing evidence does not allow for definitive conclusions on whether participation in job-related training fosters literacy skills. This is because a positive relationship between training and literacy may arise for two different reasons. On the one hand, it may indeed reflect a causal effect of training on literacy. On the other hand, it may reflect selection effects, whereby existing literacy skills drive the decision to participate in training (i.e., reverse causation), or it may be due to other sources of endogeneity, such as omitted third variables that influence both training and literacy skills. The extent to which the frequently reported association between training and literacy reflects these alternative (although not mutually exclusive) directions of causal influence remains a largely open question. The main reason for this knowledge gap is that unraveling this question requires longitudinal large-scale data that include objective measures of adults’ literacy skills, and these data are in short supply.

In the present study, we set out to unravel investigating the relationship between participation in job-related training and adult literacy skills using two-wave panel data from the German sample of the Programme for the International Assessment of Adult Competencies (PIAAC) and its follow-up, PIAAC-Longitudinal (PIAAC-L). Our sample comprised adults aged 16 to 65 years who had completed their initial cycle of education and were currently gainfully employed. The strength of PIAAC and PIAAC-L are the repeated high-quality measures of adult skills that allowed us to determine (a) whether job-related training had a direct effect on adult literacy skills (i.e., training effects) even after accounting for potential endogeneity (e.g., time-*constant* and time-*varying* confounders); and/or (b) whether individuals' skills were related to their decision to participate in job-related training (i.e., selection effects).

## Can job-related training foster literacy skills? Theory and evidence

### Returns to job-related training from the viewpoint of human capital theory

Most research on the returns to training has used human capital theory (HCT) as a guiding framework. Human capital refers to the full spectrum of skills, knowledge, and experiences a worker possesses, including not only job-specific or firm-specific skills but also more general skills such as literacy, on which we focus here.

According to HCT, education is an investment that increases the stock of human capital [14, 15]. The accumulation of human capital does not necessarily end with formal schooling but may continue after individuals enter the labor market through on-the-job learning or participation in job-related training. Some scholars have even argued that human capital earned through education in the first stages of life can be productive only if it is supplemented with follow-up investments in education [16].

The acquisition of new skills, and the productivity gains that accrue from these skills, are thought to be the main mechanisms through which job-related training produces wage premiums [10, 17]. The following cause–effect chain can be used to summarize the relationships between job-related training, skills, and wages stipulated by HCT:

education(e.g.,training)→skills(e.g.,literacy)→productivity→wages (1)

Although these key tenets of HCT are well established and widely accepted, empirical studies on the returns to job-related training more specifically have rarely investigated skills (or productivity) as training outcomes directly. Because data offering adequate skill measures have long been in short supply, a bulk of this research has instead focused exclusively on the monetary returns to training—that is, wages. In line with HCT, this research generally points to positive wage effects of training participation, although these effects have been found to differ depending on the type of training, the sample under consideration, and the models used. Comprehensive reviews of this literature can be found in Blundell, Dearden, Meghir and Sianesi [18]; Bassanini, Booth, Brunello, De Paola and Leuven [19]; Hansson [20]; and Haelermans and Borghans [21]. In the German context, on which our present study focused, most studies have found only small and often statistically non-significant wage effects of training participation [22–26]. However, there are some exceptions: Using data from the German Socio-Economic Panel (SOEP), Buechel and Pannenberg [27] identified a wage premium of training participation of roughly five percentage points for prime-age workers aged between 20 and 44 years. Using the same data set, Muehler, Beckmann and Schauenberg [28] found statistically significant wage effects of around six percentage points. Another recent study by Ehlert [29] using data from the German National Educational Panel Study (NEPS) found that employer-financed training led to a wage increase of roughly one percent.

In international comparison, training effects on wages in Germany appear to be small [25]. There are two possible explanations for this. First, there is a tight coupling between the labor market and the educational system in Germany. Germany’s “dual apprenticeship” system, which combines traditional schooling with on-the-job training in firms, is highly standardized and aims at providing job- and occupation-specific skills [7, 30]. Relative to other countries, the labor market rewards formal educational credentials obtained in this system more strongly than it rewards later (non-formal or informal) job-related training, thus tempering wage returns to training. Second, collective bargaining is comparatively strong in Germany, which leads to a compressed wage structure and might decrease returns to training because it leaves little room for individual wage growth [31].

### Effects of training on skills

Notwithstanding the significance of childhood and adolescence as sensitive periods for skill development, there is evidence that skills do continue to develop to some extent in adult life. The typically observed age profile of skill development shows a positive peak around the age of 35 years, followed by a gradual decline [2, 32]. Given the longstanding dearth of data offering adequate skill measures, only a few studies have investigated the possible effects of training on this development. These studies pursued one of two strategies. The first line of research investigated the relationship between training and skills within selective subpopulations, typically those with lower education and/or lower literacy skills. For example, Sheehan‐Holt and Smith [33] analyzed the cross-sectional association between job-related training and literacy and numeracy skills within a sample of adults with less than high school education and concluded that, although training did not impact skills themselves, it did increase the use of these skills. Using longitudinal data, Brooks, Duckett, Hutchinson, Kendall and Wilkin [34] and Brooks, Pahl, Pollard and Rees [35] used items from the International Adult Literacy Survey (IALS) and found small but statistically significant improvements in literacy after three years for adult learners. Wolf and Jenkins [36] carried out a longitudinal study assessing literacy skills at different points during participation in workplace adult literacy courses and found no evidence of improvement among native English speakers but modest, and statistically significant, improvements in literacy skills for non-native speakers. However, the authors stressed that these improvements may also have arisen from participants' longer exposure to English-speaking environments. Using data from the British National Child Development Study (NCDS), Bynner and Parsons [1] found that participation in work-related training between the ages of 16 and 37 years was positively related to functional literacy skills at the age of 37 years for those individuals, who had “poor” literacy skills at age 16. Reder [37–39] focused on a sample of adult learners without a high school (equivalency) credential and found additional evidence that participation in training increased the *use* rather than the *levels* of skills, which might translate into higher skill levels in the long run. However, as noted, all studies from this first line of investigation refer to low-skilled or low-educated subpopulations. Thus, their findings cannot be readily generalized to the overall population, where higher or lower training effects on literacy skills may be found.

Further evidence for a positive link between training and literacy skills comes from a second line of investigation that has analyzed the relationship between training and literacy skills in representative, large-scale samples of the adult population. Since the 1990s, several survey programs—namely, IALS (1994, 1998), the Adult Literacy and Lifeskills Survey (ALL; 2003, 2008), and the Programme for the International Assessment of Adult Competencies (PIAAC; 2012)—have offered objective measures of adults skills in the international context. By surveying representative samples of the working-age population (aged 16–65 years) in each participating country, these studies overcame the problem of small and selective samples and included extensive skill measures that satisfied high psychometric standards (objectivity, reliability, validity). Cross-sectional results of the 2012 PIAAC study showed large differences (ranging between 0.2 and 0.5 *SD*) in skills across all countries between individuals who had participated in training and those who had not [8]. Furthermore, based on the same PIAAC data, Sgobbi [11] and Cegolon [10] showed that training participation and skills were significantly positively related, even when formal education was controlled for.

### The question of causality

Although the few existing studies on skill returns to training suggest that there is a positive association between training and literacy, it is not yet clear whether this can be interpreted as a causal effect of training. This is because a positive relationship between job-related training and literacy skills may reflect mainly two opposing (although not mutually exclusive) causal directions. On the one hand, it may reflect causal effects of training participation on skills. According to this view, which is grounded in HCT and consistent with practice engagement theory (see, e.g., [38]), job-related training may allow the acquisition (or maintenance) of literacy skills, even though this may only be an unintended (albeit desirable) spillover effect of training designed to foster more job-specific or firm-specific skills. On the other hand, the relationship may reflect selection effects—that is, individuals with higher skills may be more likely to participate in training, either because they possess characteristics (e.g., learning motivation) that encourage them to learn or because their higher-skilled jobs require more training [8]. This would be an instance of reverse causality, in that our outcome of interest (i.e., literacy skills) actually has an effect on our treatment of interest (i.e., training participation). A sizeable number of studies have revealed a strong relationship between educational attainment and adults’ training participation [18, 23, 40, 41]. In a recent paper, Kramer and Tamm [42] were even able to identify a causal impact of initial educational investments and job-related training in adult life. If one conceives of education and literacy as closely related measures of human capital, these studies give purchase to the view that literacy-based selection effects may exist.

In addition to selection based on existing literacy skills, there may be further variables that influence both the treatment and the outcome. If these observed variables (e.g., education, gender, or age) or unobserved variables (e.g., motivation or educational preferences) are not accounted for in analytical model (i.e., are omitted), the association between training and literacy may be spurious and not reflect the true causal effect of training.

Reverse causality and omitted variable bias both pose the problem of endogeneity. Endogeneity implies that training participation cannot be viewed as exogenous in our model but is itself dependent on literacy skills and/or other (observed or unobserved) factors that drive both training and skills. In order to overcome the problem of endogeneity, some previous studies on training effects on wages have used panel data methods such as fixed effects (FE) to control for time-*constant* (fixed) omitted variables [23, 26, 40]. Another approach to identifying causal effects of training on wages while taking into account time-*varying* unobserved factors and reverse causality is to use an instrumental variable (IV) approach (see, e.g., [24, 43]). Further approaches dealing with the issue of selection into training are Heckman selection models [40], matching procedures [28], and control-group approaches [22, 41]. However, to the best of our knowledge, none of the previous large-scale studies on skills (as opposed to wages) as an outcome of training has applied such causal identification strategies. As noted earlier, most studies to date on training and skills have only been cross-sectional, as panel data on individual literacy skills are in very short supply. Moreover, finding proper instruments for measuring training is rather difficult. To the extent that they could not resolve the potential endogeneity of training, previous studies reporting a positive link between job-related training and literacy have been unable to fully unravel the question whether job-related training really does have a causal effect on literacy.

## The present study

The aim of the present study was to unravel the positive relationship between job-related training and literacy skills reported in previous studies [8, 10, 11]. Our guiding question in this regard was whether this relationship signifies that training participation fosters literacy skills (i.e., training effects) or whether it, reflects the higher propensity of more high-skilled individuals to participate in further training (i.e., selection effects or reverse causation) and/or the presence of confounding factors that influence both training participation and literacy (i.e., omitted variable bias).

We used the unique two-wave data on adult literacy skills and training participation offered by two recent large-scale studies, namely PIAAC and its German follow-up study, PIAAC-L [44, 45]. These data allowed us to overcome several limitations of the existing literature on training and literacy skills. First, the PIAAC sample comprises individuals aged between 16 and 65 years who were randomly selected from local population registers in randomly selected municipalities across Germany. This ensured that our target population of individuals who had completed their initial cycle of education and were gainfully employed was adequately represented by the sample. In contrast to the often small and selective samples used in some previous research (e.g., high-school dropouts; [46]), these data cover the entire range of literacy skills from very elementary to high and do not suffer from restriction on range, which might temper the possible effects of training on literacy skills. Second, the data include objective, and high-quality repeated measures of literacy skills that were assessed twice, in 2012 and 2015. This two-wave design allowed us to apply a comprehensive threefold causal identification strategy to probe potential training effects on literacy skills (for details, see the Method section). This threefold strategy comprised a lagged-dependent variable (LDV) specification, which makes use of the two-wave design by controlling for individuals’ previous levels of literacy and estimating the effect of training on skill development over time (i.e., residual changes in skills). In addition, the panel structure allowed us to estimate an FE model that nets out any observed or unobserved time-*constant* individual characteristics that may bias the estimate. However, FE methods solve neither the problem of time-*varying* omitted variables nor of reverse causality. Therefore, we additionally applied an IV model that addresses the latter issues. By approaching potential training effects from these three different analytical angles, and by tackling the problem of endogeneity, our threefold estimation strategy brought us closer to disentangling whether there is a causal effect of training on literacy skills or whether the previously reported associations reflect endogeneity (i.e., are due to reverse causation or omitted variable bias).

Finally, the two-wave data allowed us to explicitly test for potential selection effects of literacy on *subsequent* training participation while controlling for a large number of individual and job characteristics. As noted earlier, a host of studies have demonstrated that participation in job-related training depends crucially on individual characteristics. Almost all of these studies have identified formal educational attainment as one of the strongest predictors of adults’ training participation [18, 23, 40, 41], and this effect is causal in nature [42]. Because both education and literacy skills are closely related measures of human capital and general learning ability, it seems plausible that literacy may also influence selection into training. However, previous studies have rarely examined selection into training based on literacy skills, let alone the question of whether the relationship between literacy and subsequent training participation remains statistically significant after controlling for formal educational qualifications and other individual and job characteristics.

## Method

### Data and sample definition

The PIAAC study was conducted in 2012 and provides internationally comparable data on the skills of the working-age population (aged 16–65 years, residing in private households) across a large number of (mainly OECD) countries [8]. In Germany, a registry-based sampling design was implemented and 5,465 interviews were achieved [47]. PIAAC-L was a follow-up to the PIAAC study in Germany and comprised three additional waves of assessment conducted in 2014, 2015, and 2016. By combining PIAAC-L with PIAAC, we obtained a unique large-scale data set offering repeated, and high-quality, measures of adult skills, which made it particularly suitable for the purposes of our study (for further information see S1 Appendix).

The assessment of literacy skills took place in PIAAC 2012 and PIAAC-L 2015. Although information on participation in job-related training was collected in all waves of PIAAC-L, the 2015 wave included only one question about participation in training and thus yielded insufficient information for our present intent. Therefore, we used data from PIAAC 2012 as our baseline measurement for training and literacy skills (t_1_). We combined information on training from PIAAC-L 2014 and on literacy skills from PIAAC-L 2015 for our second measurement (t_2_). Combining data from 2014 and 2015 ensured that participation in training took place *before* the second skill assessment, a necessary condition to establish training effects on skills. Table 1 gives an overview of the measures available in the different waves.

**Table 1:** Measures used from different waves of PIAAC/PIAAC-L.

	PIAAC 2012	PIAAC-L 2014	PIAAC-L 2015
Literacy skills	X		X
Training information (12 months preceding the survey)	X	X	
Sociodemographic and job characteristics	X	X	X

A total of 3,263 respondents participated in the waves under consideration in the present study (i.e., PIAAC 2012 and the 2014 and 2015 waves of PIAAC-L). We excluded individuals who were still in their initial cycle of education at the time of the survey. Furthermore, we excluded respondents whose native language was not German, because PIAAC measures skills in the language of the country of residence. Thus, for non-native German speakers these measures actually represented foreign language skills (rather than literacy skills).

Finally, we restricted our sample to employed individuals. We did so for several reasons. First, the focus of our study was on the effects of job-related training, because it is the form of training that is most frequently used by working-age adults [6]. Although unemployed individuals are also able to participate in some forms of job-related training, the types of training in which they participate are likely to differ markedly from those typically undertaken by employed individuals. Second, the data provide information on job characteristics for employed individuals only. Therefore, restricting the sample allowed us to analyze further characteristics that might drive selection into training. After applying these restrictions, there were 1,773 individuals in our full sample, and we had complete information on all job characteristics for 1,506 of these individuals. Table 2 reports basic statistics on individual characteristics for the full sample and the subsample for which we had further job characteristics. The two samples do not differ markedly in the distribution of these characteristics.

**Table 2:** Sample characteristics (T_2_).

	Total analysis sample (*N* = 1773)			Additional job characteristics available *(N =* 1506*)*		

Statistic	Range	*M*	*SD*	Range	*M*	*SD*
Age in years	18–68	46	10.6	18–68	45.3	10.4
Female (%)		46.0%			47.2%	
Potential experience in years	0–51	26.9	11.13	0–51	26.4	10.9
Formal qualification (%)						
ISCED 1/2		3.8%			3.7%	
ISCED 3/4		57.0%			57.7%	
ISCED 5B		12.2%			11.8%	
ISCED 5A/6		27.0%			26.7%	
Literacy skills (mean)	0–500	284.2	42.8	0–500	284.4	42.9
Correlation literacy t1 & t2		0.83			0.83	
*Job characteristics*						
Full-time employed (%)					75.4%	
Part-time employed (%)					24.6%	
Tenure in years				0–48	13.2	10.8
Complex problem solving (at least once a week; %)					42.0%	
Computer use (at least once a week; %)					77.3%	
Firm size (%)						
small					20.1%	
medium					29.8%	
large					50.1%	
Participation in training during the 12 months preceding the survey (%)		58.5%			57.9%	

### Measures

#### Participation in training

The operationalization of job-related training differed slightly between the waves. PIAAC 2012 contains information on respondents’ participation in different types of training activities in the 12 months preceding the survey, irrespective of whether they participated for job-related or non-job-related reasons. The types of training activities are as follows: 1) open or distance education; 2) organized sessions for on-the-job training or training by supervisors or co-workers; 3) seminars or workshops; 4) private lessons. For each type of training activity, participation in job-related training was coded as a binary variable with “1 –participation in job-related training” if one of the following conditions held: (a) respondents stated that the primary reason for participating in training was job-related; (b) respondents stated that the training took place during working hours; or (c) respondents stated that the training was conducted by a supervisor or by colleagues. In PIAAC-L, respondents were asked directly whether they had participated in any type of job-related training in the year preceding the survey.

#### Literacy skills

PIAAC 2012 and PIAAC-L 2015 both included measures of literacy, which enabled us to analyze changes in these skills over time. The literacy skill scores for each person were obtained using an item response theory (IRT) model. In order to interpret the differences between the scores for any single person as differences in the measurement, the IRT model set the scores from the two measurements in 2012 and 2015 on the same latent scale (for more information, see Carstensen, Gaasch and Rothaug [48]). The results of the literacy assessment are given on a scale ranging from 0 to 500 with a mean of 250 points and a standard deviation of 50 points. To facilitate interpretation, the continuous scale can be categorized into five skill levels. For further details on the skills assessment in PIAAC and PIAAC-L, see Supporting Information.

#### Control variables

We controlled for a broad range of individual and job characteristics to avoid omitted variable bias and to control for heterogeneity between the respondents. All of our models controlled for gender (1 = *female*, 0 = *male*), potential work experience (age in years minus 6 minus years of education) in years and its square. We included formal educational attainment with four levels according to the International Standard Classification of Education 1997 (ISCED 1997): ISCED 1/2; ISCED 3/4; ISCED 5A/6; ISCED 5B.

Job characteristics included working time (1 = *full-time*, 0 = *part-time*), tenure in years and its square. Furthermore, we included two binary proxies for job content: computer use at work (1 = *yes*, 0 = *no*) and complex problem solving at work (1 = *at least once a week*, 0 = *less than once a week*) and firm size (*small*, *medium*, *or large*). The comparability of the latter variable between t_1_ and t_2_ is limited. At t_1_, a small firm was defined as having 1–10 employees, a medium-sized firm as having 11–250 employees, and a large firm as having more than 250 employees. At t_2_, a small firm was defined as having 1–10 employees, a medium-sized firm as having 11–200 employees, and a large firm as having more than 200 employees. We included the current occupational status in four groups (“skilled occupations,” “semi-skilled white-collar occupations,” “semi-skilled blue-collar occupations,” and “elementary occupations”) and the current industry via 21 dummies for the International Standard Classification of Industries (ISIC). In addition, our models contained a dummy indicating whether a respondent lived in eastern Germany or western Germany, because previous research has found differing training participation rates and effects in the two regions [27].

### Data analytic strategy

We unraveled the relationship between job-related training and individual literacy skills in two main analytical steps. In the first set of models, we examined whether participation in training contributed to changes in literacy. To probe such potential training effects on literacy skills, we applied a variety of different specifications, including ordinary least squares (OLS), LDV, FE, and IV models. Each of these specifications approaches potential training effects from a slightly different analytical angle, and each has its own unique strengths and weaknesses, as we outline below. By combining these different specifications, we obtained a comprehensive picture of the relationship between training participation and literacy skills and got as close as possible to identifying potential causal effects of training. In the second set of models, we examined selection effects, testing whether individual literacy skills predicted subsequent participation in job-related training and whether this relationship held after controlling for sociodemographic and job characteristics (especially formal educational attainment).

#### Training effects on literacy skills

The first set of models estimated potential effects of training participation on literacy skills. In a first step, we estimated OLS models regressing literacy skills at t_2_ on job-related training in the two years preceding the study. These models give a first and easy-to-interpret indication of the relationship between training and literacy when controlling for the set of job characteristics variables described above:

$${Literacy}_{i,t2}=\alpha_{0}+\alpha_{1}TR_{i,t2}+\beta X_{t2}+e_{i,t2}$$ (2)

where *i* indicates the individual and t denotes the time. *TR* is a dummy variable that equals 1 if an individual participated in training in the preceding year and 0 otherwise, ***X*** is a vector of control variables and *e* is an idiosyncratic error term. Coefficient *α*_1_ and coefficient vector ***β*** are parameters to be estimated; they give the effects of training and of the control variables on literacy.

In our next specification, we made use of the two-wave panel structure of our data and additionally included a lag of literacy skills (i.e., controlled for literacy at t_1_). In contrast to the OLS specification, this LDV specification makes use of the repeated measures of literacy, relating job-related training to subsequent (residual) *changes* in literacy skills over three years.

$${Literacy}_{i,t2}=\alpha_{0}+\alpha_{1}TR_{i,t2}+\alpha_{2}{Literacy}_{i,t1}+\beta X+e_{i,t2}$$ (3)

The parameters in Eq (3) are defined in the same way as in Eq (2). The additional coefficient *α*_2_ gives the association between literacy at t_2_ and t_1_. The LDV model is more informative than the OLS model because it controls for potential selection into training of respondents based on their literacy skills (i.e., the t_1_ literacy value). Moreover, the LDV model correctly models the temporal ordering of training and *subsequent* literacy scores. However, the LDV model is still subject to the problem of endogeneity that may arise either from time-*constant* or time-*varying* omitted variables that influence *both* training participation and literacy or from reverse causality. As such, it does not allow for causal inferences regarding training effects. Therefore, in a third step, we applied an FE model. This model controls for any (observed and unobserved) time-*constant* factors that might be related to both literacy and training and might bias the OLS or LDV estimates of their relationship. These factors are canceled out when applying the FE (or within) transformation, that is

$${Literacy}_{i,t}-{\bar{Literacy}}_{i}=\alpha_{1}(TR_{i,t}-{\bar{TR}}_{i})+\beta {(X}_{i,t}-{\bar{X}}_{i})+u_{i,t}-{\bar{u}}_{i}$$ (4)

where ${\bar{Literacy}}_{i},\;{\bar{TR}}_{i},\;{\bar{X}}_{i},$ and ${\bar{u}}_{i}$ are the averaged values of skills, training, the control variables, and the error term respectively.

Whereas the FE model eliminates potential biases due to time-*constant* confounders, it solves neither the problem of time-*varying* unobserved variables nor the problem of reverse causality. Thus, FE estimates may still be biased if changes in literacy cause changes in training participation, or if time-*varying* factors (e.g., childbearing, health issues) influence both literacy and training participation.

In order to overcome the limitations of the FE approach, we additionally estimated an IV model, which tackles the problem of endogeneity in a different way. The key idea behind the IV approach is that the treatment variable (in our case, training) consists of two parts: One is subject to endogeneity, the other is not. The IV approach aims to isolate the latter part of the treatment variable by using only the part of the variation in the treatment that can be attributed to a third variable, the instrument. The instrument is an additional variable that must satisfy certain conditions: First, it must be correlated with the endogenous variable, namely training. This condition is called “instrument relevance.” Second, it must be exogenous, which means that it should not be correlated with the outcome (in our case, literacy skills)—apart from the indirect effect channeled through the treatment variable (in our case, individual training participation). This condition is called “instrument exogeneity” or “exclusion restriction.” The first condition can be simply tested empirically by regressing the treatment on the instrument, whereas the second conditions cannot be tested, as this would require testing the correlation between the instrument and the error term, which is unobserved.

The literature on training effects has proposed only a few instruments that are likely to meet these assumptions. Kuckulenz and Maier [43] and Kuckulenz and Zwick [24] suggested using average training participation rates per industry. The core of their idea is that individuals who are employed in an industry with a high training intensity are more likely to participate in training (instrument relevance). The only way in which training intensity in an industry can influence literacy skills should be via individuals’ own training participation (instrument exogeneity). PIAAC gives information on the industrial sector (ISIC) of each individual, which we merged with information on the average training participation per industry obtained from the German Microcensus 2012 [49]. Because the instrument is available only for 2012, we report only the results of a cross-sectional IV specification. Eq (5) gives our first-stage specification. It includes average training participation per industry as our instrument (I) and the same set of covariates ***X*** as above. Eq (6) refers to the second stage where $\hat{TR}$ is the predicted value of actual training participation obtained from the first stage regression and *λ* can be interpreted as an unbiased effect of training on literacy:

$$TR_{i}=\delta_{0}+\delta X+\theta \;I_{i}+r_{i}$$ (5)

$$Literacy_{i}=\rho_{0}+\rho X+\lambda \hat{TR_{i}}+v_{i}$$ (6)

This instrument is subject to possible limitations. If there are any other causal pathways through which average training participation per industry influences individuals’ literacy skills (i.e., if the “exclusion restriction” does not hold), our IV estimates may be biased. Nevertheless, our IV models served as a useful addition to our other modeling approaches with regard to causal identification. By viewing the effects from different modeling perspectives, we sought to maximize the confidence in our inferences concerning the effects of training on literacy skills.

#### Selection into training

While the above training effect models aimed to *eliminate* potential bias due to self-selection into training, our second set of models explicitly probed such selection effects in order to quantify them. We applied a probit model in which we regressed training (*TR*) on literacy skills, formal education, and further sociodemographic and job characteristics (summarized under ***X***) that previous studies have commonly identified as important drivers of training participation [50]. We used data from t_1_ for literacy skills and from t_2_ for training in order to ensure that skills were measured *before* training participation. G(.) is a standard normal cumulative distribution function yielding the probit model:

$${Pr(TR}_{i}=1|X)=G(\beta X)$$ (7)

To gauge the relative contribution of education and literacy skills for selection into training, we estimated three models with different sets of predictors. The first model included only literacy skills as a measure of human capital. The second model included both literacy skills and formal educational qualifications. In a third model, we included further job characteristics.

#### Cross-validation with wages

In addition to our training effects models in which literacy skills were the outcome, we estimated the same set of models (OLS, LDV, FE, and IV) with wages as the dependent variable. We used the log of self-reported gross hourly wages in euros and trimmed the bottom and top one percent of the wage distribution to limit the influence of outliers. Because information on wages was not available for all individuals, we had a limited sample size of 1315 (with job characteristics: 1145) individuals in our wage models.

Our motivation behind these models was replication and cross-validation. Because to date there have been no large-scale studies estimating training effects on literacy skills with repeated-measures data, we could investigate whether our current data and measurement instruments yielded results concerning the wage premiums of training that are comparable to those of previous studies. If the wage premiums turned out to be comparable to those reported in previous studies, this would help rule out that our findings concerning literacy skills were driven by idiosyncrasies of our sample or our measures of training.

## Results

### Descriptive statistics on literacy skills

Before turning to our main research questions, we present descriptive statistics on literacy skills that are of general interest to literacy research and that prepare the ground for our multivariate analyses. Specifically, we analyzed (1) the rank-order stability of literacy skills over three years; (2) average changes in literacy skills by age; and (3) differences in literacy skills according to participation in job-related training.

First, we asked how stable or malleable literacy skills were during adulthood in terms of rank-order stability—a question that can be answered conclusively only with longitudinal data. A very high rank-order stability (e.g., in excess of *r* = .90) would imply that training effects on changes in literacy were unlikely. In this regard, Table 2 shows a correlation of *r* = .83 between literacy at t_1_ and literacy at t_2,_ indicating a high, but far from perfect, temporal consistency of literacy skills over the three-year period.

**Fig 1:**
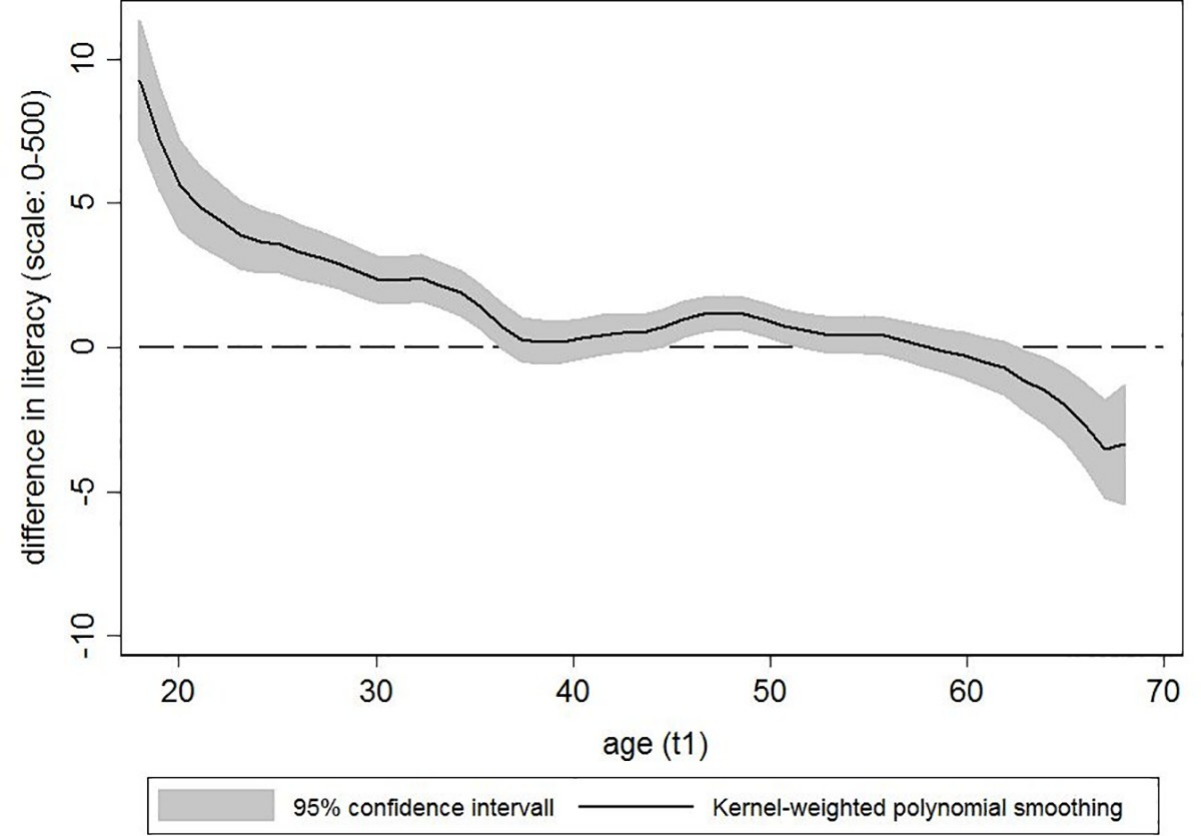
Changes in literacy skills over three years by age.

Second, Fig 1 shows the average *changes* in literacy skills across the three-year study period by age at t_1_. This figure is based on the difference score between t_1_ and t_2_ literacy. In line with previous cross-sectional findings [2, 32], we observed small average increases in literacy skills over three years among respondents aged 35 years or younger. This was followed by a mid-life plateau until age 60 during which little change in literacy occurred. Among respondents aged 60 years and older, we observed average declines in literacy over the three-year period. However, both the average gains among young adults and the average losses among older adults were small, amounting to less than 10 scale points.

Third, and more important for our present intent, there were descriptive differences in literacy skills by training participation. When comparing the average literacy skills of individuals in our sample who had participated in training in the 12 months preceding the survey to individuals who had not participated in training, we found a skill gap of 19 points (0.38 *SD*). This is in line with previous cross-sectional results from PIAAC, showing—across countries—that individuals who had participated in training had higher literacy levels than individuals who had not participated in training [6, 8, 10, 11]. Although the size of this difference was considerable, the descriptive results did not answer the question of whether this gap stemmed from selection into training of more skilled individuals or whether there was indeed a positive causal effect of training on literacy. This is the question to which we now turn in our multivariate models.

### Effects of training on literacy

Table 3 gives the results of our OLS and LDV specifications testing potential training effects on literacy. Results in Column A suggest that there was a positive and statistically significant relationship between training and literacy skills in the OLS specification. Individuals who participated in training showed a subsequent skill advantage of seven points (the equivalent of 0.14 *SD*) over those who did not. This advantage dropped to roughly six points (0.12 *SD*) once we included further job characteristics in Column B, but it remained statistically significant. Columns C shows the results of our LDV specification, which additionally included a lag in literacy skills to estimate the effect of training on (residual) changes in literacy over three years. Compared to Column B, the association between training and literacy skills decreased to two points (0.05 *SD*) once we included literacy at t_1,_ and it was no longer statistically significant. This suggests that training participation does not lead to (residual) changes in literacy skills. Further results showed that formal educational qualifications had a positive and statistically significant effect on literacy, even when job characteristics were controlled for. This effect vanished once we included the lag in literacy in Column C. To allow for potential heterogeneity of training effects across subgroups, we estimated an average treatment effect model including interaction between training and all covariates. We did not find any significant differences between different groups of individuals. The results are available upon request.

**Table 3:** Training effects–OLS and LDV models.

	Literacy Skills			Log Hourly Wages		
	A	B	C	D	E	F
Training	7.074**	5.689*	1.794	0.118***	0.042*	0.026
	(2.335)	(2.485)	(1.866)	(0.024)	(0.021)	(0.017)
**Education** **(Reference: ISCED 2 or lower)**						
ISCED	28.722***	22.015**	−3.856	0.032	−0.146	−0.157*
3/4	(6.521)	(7.042)	(5.615)	(0.072)	(0.087)	(0.074)
ISCED	42.691***	32.063***	−1.171	0.251**	-0.064	-0.136
5B	(7.283)	(8.000)	(6.584)	(0.079)	(0.091)	(0.077)
ISCED	68.241***	53.436***	9.872	0.439***	0.010	-0.079
5A/6	(6.658)	(7.585)	(6.337)	(0.078)	(0.091)	(0.078)
Literacy				0.086***	0.030	0.019
(standardized)				(0.017)	(0.015)	(0.011)
Lag in			0.763***			
Literacy			(0.024)			
Lag in						
Wages						0.517***
						(0.030)
Female	2.259	−2.424	−3.394	−0.187***	−0.155***	−0.115***
	(2.151)	(2.853)	(2.166)	(0.024)	(0.025)	(0.020)
Experience	0.113	−0.334	−0.068	0.030***	0.025***	0.005
in years	(0.409)	(0.475)	(0.317)	(0.005)	(0.004)	(0.004)
Experience	−0.017*	−0.007	−0.003	−0.000***	−0.000***	−0.000
squared	(0.008)	(0.010)	(0.007)	(0.000)	(0.000)	(0.000)
Job		included	included		included	included
characteristics						
Constant	248.061***	249.500***	60.102***	2.265***	2.346***	1.543***
	(8.214)	(12.721)	(12.093)	(0.083)	(0.130)	(0.131)
R^2^	0.277	0.330	0.724	0.332	0.556	0.686
*N*	1,773	1,506	1,506	1,315	1,145	1,145

Job characteristics include full-time employment, tenure, tenure squared, firm size, a dummy variable indicating a large amount of complex problem solving at work, a dummy variable indicating the use of computers at work, four occupational dummies, 21 industry dummies, and a dummy variable indicating whether respondents lived in eastern Germany. Standard errors in parentheses.

* p < 0.1

** p < 0.05

*** p < 0.01.

The results from our OLS and LDV models give a first good insight into the relationship between job-related training and literacy. However, they do not take into account the problem of endogeneity (either due to reverse causality or omitted variables). Thus, they do not allow for causal interpretations. Our FE and IV models addressed this problem. The FE model accounts for time-*constant* effects that might drive the positive relationship between training and literacy. The IV approach addresses the problems of time-*varying* omitted variables and reverse causality (i.e., literacy skills affecting the decision to participate in training).

Table 4 presents the results obtained from the FE model. We report only the full specification including job characteristics. In this model, training had a very small (0.02) and statistically non-significant relationship to (changes in) literacy skills. In line with the LDV specification, the FE specification rejects the hypothesis of a causal effect of training on literacy.

**Table 4:** Training effects–FE models.

	Literacy Skills	Log Hourly Wages
	A	B
Training	0.019	0.012
	(1.745)	(0.016)
**Education** **(Reference: ISCED 2 or lower)**		
ISCED 3/4	3.614	0.560***
	(5.923)	(0.159)
ISCED 5B	−6.301	0.739***
	(8.770)	(0.183)
ISCED 5A/6	−0.886	0.788***
	(8.665)	(0.187)
Experience	0.597	0.051***
in years	(0.591)	(0.007)
Experience	−0.014	−0.001***
squared	(0.011)	(0.000)
Job characteristics	included	included
Constant	281.932***	1.189***
	(12.974)	(0.235)
R^2^	0.037	0.250
*N (persons)*	1506	1145
*N (person-period observations)*	3,006	2,290

Job characteristics include full-time employment, tenure, tenure squared, firm size, a dummy variable indicating a large amount of complex problem solving at work, a dummy variable indicating the use of computers at work, four occupational dummies, 21 industry dummies, and a dummy variable indicating whether respondents lived in eastern Germany. Standard errors in parentheses.

* *p* < 0.1

** *p* < 0.05

*** *p* < 0.01.

Finally, results for our IV approach can be found in Table 5. Again, we report only the full specification including job characteristics. The lower part of the table gives the results from our first-stage estimation and shows that average training participation per industry had a positive and statistically significant effect on individual training participation. Thus, the assumption of “instrument relevance” is met. Although the F-test statistic of 27 is rather low, it is still above commonly used thresholds of 10 and suggests that we do not have a weak-instrument problem [51]. After instrumenting for selection into training, we found no statistically significant effect on literacy. This confirms our previous results from the LDV and FE models: After controlling for individual and job characteristics and taking endogeneity into account, job-related training had no causal effect on literacy. As stated before, our instrument is available only for 2012, which limited our analyses to t_1_. In order to compare the IV and OLS results directly, Table 5 additionally gives the OLS results for t_1_ (2012). These results do not differ substantially from the OLS results for t_2_ (2015) reported in Table 3.

**Table 5:** Training effects–instrumental variables approach.

	Literacy Skills		Log Hourly Wages	
	OLS	IV	OLS	IV
	A	B	C	D
Training	2.191	20.370	0.100***	0.152
	(2.400)	(23.232)	(0.022)	(0.230)
**Education** **(Reference: ISCED 2 or lower)**				
ISCED 3/4	28.123***	25.297***	0.152***	0.148
	(6.954)	(7.830)	(0.082)	(0.095)
ISCED 5B	36.930***	32.624***	0.330***	0.323***
	(7.908)	(9.079)	(0.089)	(0.110)
ISCED 5A/6	48.106***	44.150***	0.496***	0.483***
	(7.345)	(8.958)	(0.089)	(0.108)
Female	−3.021	−3.455	−0.115***	−0.111***
	(2.606)	(2.724)	(0.026)	(0.030)
Experience	−0.527	−0.787	0.028***	0.030***
in years	(0.439)	(0.535)	(0.005)	(0.006)
Experience	−0.003	0.003	−0.001***	−0.001***
squared	(0.010)	(0.127)	(0.000)	(0.000)
Job characteristics	included	included	included	included
Constant	270.176	269.618***		1.794***
	(8.873)	(9.043)		(0.121)
R^2^	0.256	0.208	0.537	0.536
**First Stage**				
Agg. training participation		0.575***		0.710***
per industry (ISIC 3 digit)		(0.135)		(0.159)
F-Test		18.12***		20.07***
R^2^		0.139		0.126
*N*	1,506	1,506	1,145	1,145

Job characteristics include full-time employment, tenure, tenure squared, firm size, a dummy variables indicating a large amount of complex problem solving at work, a dummy variable indicating the use of computers at work, four occupational dummies, 21 industry dummies, and a dummy variable indicating whether respondents lived in eastern Germany. Standard errors in parentheses;

* *p* < 0.1

** *p* < 0.05

*** *p* < 0.01

### Selection into training

Table 6 gives the results of our selection models and shows average marginal effects computed from the probit models. Our main interest in these models is whether literacy skills predicted subsequent training participation––and whether they did so incrementally over other factors that may have driven selection into training.

**Table 6:** Selection into training.

	A	B	C
Literacy skills (standardized)	0.077***	0.037*	0.035*
	(0.013)	(0.015)	(0.014)
**Education**			
**(Reference: ISCED 2 or lower)**			
ISCED 3/4		0.056	-0.035
		(0.064)	(0.064)
ISCED 5B		0.219**	0.023
		(0.074)	(0.077)
ISCED 5A/6		0.280***	0.095
		(0.071)	(0.077)
Female	0.019	0.032	0.009
	(0.025)	(0.025)	(0.030)
Experience	0.006	0.006	0.009
in years	(0.005)	(0.005)	(0.005)
Experience	-0.000*	-0.000*	-0.000*
squared	(0.000)	(0.000)	(0.000)
Job characteristics			included
Constant	0.402***	0.245**	0.129
	(0.059)	(0.085)	(0.151)
R^2^	0.026	0.047	0.126
*N*	1,773	1,773	1,506

The results reported here are the average marginal effects obtained from the multivariate Probit model described by Equation (7). Job characteristics include full-time employment, tenure, tenure squared, firm size, a dummy variable indicating a large amount of complex problem solving at work, a dummy variable indicating the use of computers at work, four occupational dummies, 21 industry dummies, and a dummy variable indicating whether respondents lived in eastern Germany. Standard errors in parentheses.

* *p* < 0.1

** *p* < 0.05

*** *p* < 0.01.

Results in Column A show that individuals with a one standard deviation higher literacy score were eight percentage points more likely to participate in job-related training than individuals with an average level of literacy skills. This effect shrank to roughly four percentage points once we included a measure of formal educational qualification in Column B. In addition, we found a substantial effect of educational attainment: Respondents with the highest educational level were 28 percentage points more likely to participate in training compared to their lower-educated counterparts. This result is in line with previous research, which identified education as the main determinant of training participation [40–42]. Once we controlled for further job characteristics in Column C, the effect of education was no longer statistically significant. However, the effect of literacy skills remained similar in size to that in Column B.

Further results show that neither gender nor age or work experience seem to have driven participation in job-related training. With regard to job characteristics (results not shown), we found higher rates of training participation among individuals working in large firms (compared to small or medium firms), among for individuals who used a computer at work, and among individuals who worked in skilled occupations (compared to semi-skilled or elementary occupations). These findings are in line with previous studies on the determinants of training (see, e.g., [23, 24, 50]).

In sum, these models demonstrate that selection into training depended on individual and job characteristics. Crucially, they show that individuals with higher literacy were more likely to participate in job-related training, even after taking account formal education and other factors.

### Additional analyses: Training effects on wages

How do the effects of training on literacy skills (or, more precisely, the lack of effects) compare to those on wages? Tables 3–5 also include the results of the OLS, LDV, FE, and IV models for log hourly wages.

In the OLS model in Columns D and E of Table 3, participation in training was related to an increase of 12 percentage points in log hourly wages, which dropped to four percentage points when job characteristics were included. This is in line with previous cross-sectional analyses of training effects on wages (e.g., [28]). The specification in Columns F additionally included a lag in log hourly wages to take account of state dependence in wages. When the previous level of wages was controlled for, we did not find a statistically significant positive relationship between training participation and (residual changes in) wages.

All our wage specifications included a standardized measure of literacy skills in the regressions to show that the wage premium of training participation found in previous studies held even after controlling for literacy. Moreover, in our OLS model we found that literacy skills showed a statistically significant positive association with wages, thereby demonstrating that there were returns to literacy skills that existed independent of educational credentials (and training participation). This is in line with Hanushek, Schwerdt, Wiederhold and Woessmann [52], who found positive effects of these skills on wages across countries, even when formal education was controlled for.

Column B of Table 4 contains the wage results for the FE model. As in the case of literacy, only the full specification including job characteristics is reported. We found that job-related training had a very small (0.012) and statistically non-significant effect on wages, thus rejecting the hypothesis of causal training effects. The same conclusion emerged from our IV model in Table 5, where we did not find a statistically significant effect of training on wages.

To sum up, our OLS models suggested a positive association between training and hourly wages. From our FE and IV models, which took account of endogeneity of training, we concluded that this positive relationship did not reflect a causal effect. Thus, we found essentially the same pattern of (null) results for wages as for literacy skills as an outcome. Moreover, our findings are consistent with the majority of studies conducted in Germany, which have revealed that the wage returns to training are small or non-existent [22, 24–26, 29]. Larger wage returns to training have been found only in a few studies, yet these diverging results may be explained by the fact that these studies focused on specific age groups (i.e., 20-44years; [27]) or a specific form of training (i.e., general training; [28]). The close correspondence between our findings and previous ones also enhances confidence in our findings concerning literacy; if results regarding wages are similar to previous findings, it seems highly unlikely that our findings regarding literacy skills are driven by idiosyncrasies of the current sample, training measure, or analytical approach.

## Discussion

Can participation in job-related training contribute to the formation and maintenance of adults’ literacy skills? Frequently reported associations between participation in job-related training and higher literacy skills [8, 10, 11] suggest that this is the case. However, as we argued, this association between training and literacy may reflect more than one of several equally plausible (and not mutually exclusive) directions of influence: On the one hand, job-related training—even types of training intended to impart highly job-specific or firm-specific skills—may indeed exert positive spill-over effects on the development of literacy skills (i.e., training effects). On the other hand, workers may select themselves into job-related training based on their level of literacy skills, whereby those with higher levels of skills are more likely to participate in further training (i.e., selection effects). In addition to selection based on literacy skills, further sources of endogeneity such as omitted third variables that influence both training participation and literacy skills may bias estimates of purported training effects. In the present study, we set out to unravel these possibilities, analyzing whether the association between training and literacy reflects training effects or rather selection and other sources of endogeneity. For this purpose, we used unique two-wave panel data from two representative large-scale surveys representing the German population between 16 and 65 years and offering repeated, and high-quality, measures of literacy skills. In doing so, our study is the first to analyze the relationship between job-related training and literacy using repeated-measures data *and* objective literacy measures that cover the entire range of skills from very elementary to high.

Descriptive analyses revealed the expected positive association between job-related training and literacy skills reported in previous studies, with an (unconditional) skill gap of more than one-third of a standard deviation between those who participated in training and those who did not. Even after controlling for a host of sociodemographic and job characteristics in the OLS model, training participation was linked to 7 points (0.14 *SD*) higher literacy skills. However, this relationship largely vanished once prior skill levels were controlled for in the LDV models. Likewise, no indications of causal training effects emerged from our subsequent FE and IV models. Thus, our three more rigorous identification strategies (LDV, FE, and IV)—which accounted for potential endogeneity arising from omitted variables or reverse causation in the best way possible with the current data—paint a clear picture: The association between training and literacy does not reflect a causal effect of training. Our findings can help explain why previous cross-sectional studies have often reported a positive association between training and literacy, whereas most longitudinal studies have found no effects or only small ones [33, 37–39]. Differences from those few studies that did find (small) training effects on literacy over longer periods of time [1, 34, 35] might be explained by the fact that these studies focused on low-skilled subgroups, in which a higher degree of skill development might occur.

On the other hand, our selection models revealed that literacy skills did predict subsequent training participation. Specifically, an increase of one standard deviation on the literacy scale (0–500 points) increased the likelihood of participating in training by more than seven percentage points. This selection effect held even after accounting for a broad range of other factors, including formal educational attainment *and* a host of job characteristics. Our results extend previous findings that identified formal education as the main (and causal) driver of selection into training (see, e.g., [23, 40–42]). They show that, over and above education and job characteristics, an individual’s level of literacy skills is incrementally linked to the likelihood of participating in training. This might be explained by the fact that our measures of formal education only partly capture worker’s human capital, whereas actual skills such as literacy provide additional information on the capabilities acquired or used at the workplace. Literacy might drive selection into training through several pathways. First, individuals with higher literacy skills may choose to participate in training at higher rates because their higher literacy leads to (or reflects) a higher motivation and ability to learn. Second, their higher literacy skills may allow these individuals to obtain jobs that are more complex and require more training. Employers will be more likely to invest in training for individuals with higher literacy skills who occupy more complex jobs.

In sum, then, our analyses yielded no evidence that the association between training and literacy skills reflects a causal effect of training. Instead, they suggest that the positive association between training and literacy skills in the OLS models was driven by endogeneity of training and unobserved heterogeneity that was not fully accounted for in the OLS models and in earlier studies using similar (cross-sectional) specifications. In other words, rather than participation in job-related training contributing to the development of literacy skills, workers with higher skill levels are more likely to participate in training.

There are several possible explanations for the absence of a causal effect of job-related training on literacy. First, as mentioned before, we analyzed the effects of job-related training—which is typically designed to foster job-specific skills—on broad skills, namely literacy. Although theoretical considerations (e.g., based on practice engagement theory) [46] and empirical findings highlight the possibility of such spill-over effects, this is not what we found in our data. A second explanation for the absence of training effects on literacy in our analysis might lie in the three-year time span under study. It is conceivable that skill gains might either accrue directly after training and might be too short-lived to be detectable with the data at our disposal. Alternatively, training effects might accrue only over longer time periods and after significant investments in training. The latter is in line with Reder [37–39] who found that, although training did not have immediate benefits for literacy skills, it did increase literacy skill use, which might still translate into higher skill levels in the long run.

For replication and cross-validation purposes, we also estimated potential training effects on wages. Akin to our models in which literacy was the outcome, our initial OLS results also suggested a positive effect of training. Participation in job-related training was associated with an increase of twelve percentage point in hourly wages, which dropped to 4 percent when job characteristics were controlled for. However, results from our LDV model, which controlled for previous wage levels, yielded no evidence of statistically significant training effects after controlling for a wide range of job characteristics. Furthermore, when accounting for endogeneity using an FE and IV approach, no evidence of a causal training effect on wages emerged. This is consistent with previous research concerning training effects on wages: After accounting for possible endogeneity of training, no statistically significant effects were present (see, e.g., [22, 23, 25]).

Together, our findings suggest that previous cross-sectional research has likely overestimated both the monetary returns (wages) and non-monetary returns (literacy skills) to job-related training. This is because most cross-sectional research is unable to unravel the two directions of influence—selection and training effects—from which a positive relationship between training and literacy skills or wages may arise. In order to adequately capture returns to training, future research should draw on repeated-measures designs and apply models that allow for better causal identification strategies.

### Limitations and directions for further research

Our study is subject to two main limitations that point to avenues for future research. The first concerns the two-wave design; the second relates to the measure of training.

Concerning the two-wave design of the data at our disposal, the repeated measures of literacy in PIAAC-L are clearly a significant advancement over the bulk of previous research, which has been largely cross-sectional. Indeed, ours is among the first large-scale studies on the relationship between training and literacy utilizing a repeated-measures design. Yet, it should be borne in mind that training effects on literacy might be realized after a shorter or a longer time period following training participation. Our current results apply only to the three-year period under study that was covered by the two-wave design. It would be desirable for future studies to investigate the effects of training on skills with more intensive longitudinal data offering more frequent skill assessments and covering longer time spans. Unfortunately, no such data are available yet.

Concerning the measure of training, we relied on PIAAC’s widely used training measures. Albeit relatively coarse, these measures are valuable in that they allow respondents’ often highly idiosyncratic training activities to be assessed in a parsimonious fashion. Yet there are some indications from previous studies that not only participation in training per se but also the intensity of training determines its effects [23, 53]. In addition, there might be differences according to the specific type of training (e.g., whether or not courses take place during working hours) [54]. Although more fine-grained data on training are currently in short supply and generally hard to acquire, future studies could cast further light on whether training intensity, content, and scheduling moderate potential training effects on literacy skills.

## Conclusion

The key contribution of our study is to answer the question as to whether the frequently observed (e.g., [8, 10, 11]) positive association between participation in job-related training and literacy skills reflects causation (i.e., training effects on literacy skills) or selection (i.e., effects of literacy skills on training participation), or whether it is due to other sources of endogeneity, such as omitted variable bias. In this regard, our findings suggest that selection effects dominate the picture: Individuals with higher literacy skills were more likely to participate in job-related training, even after controlling for formal education and a host of job characteristics. Contrariwise, we found no evidence of a causal effect of training on literacy skills over the three-year period under study—at least not with regard to way in which training and skills are defined and measured in the context of PIAAC. These results do not imply that job-related training has no beneficial effects whatsoever for participants and employers. On the contrary, we have taken it for granted throughout this article that job-related training contributes to job-specific or firm-specific skills—that is, idiosyncratic skills that cannot be assessed in large-scale studies such as PIAAC. If training did not successfully contribute to the formation of job-specific skills, employers would surely be unwilling to finance it and employees would be equally unwilling to participate in it. Moreover, several studies that controlled for unobserved selection and unobserved heterogeneity have shown that, under certain conditions, participation in training can lead to wage premiums or increased employment prospects. However, as far as general literacy skills are concerned, our data provide no evidence for causal training effects. In other words, literacy skill gains through specific job-related training not designed to foster literacy in the sense of a spillover effect do not appear to be likely. For policymakers and practitioners interested in fostering literacy skills, training that is specifically designed to foster such skills is likely to be a more suitable option.

## Supporting information

S1 Appendix Skills assessment in PIAAC and PIAAC–L. (PDF)
