# Supplementary material for: Self-perfection or self-selection? Unraveling the relationship between job-related training and adults’ literacy skills
Source: PLoS One. 2019 May 1;14(5):e0215971. doi: 10.1371/journal.pone.0215971 (PMC6493749; doi:10.1371/journal.pone.0215971)
Supplement: S1 Appendix — (PDF) [file pone.0215971.s001.pdf]

Skills assessment in PIAAC and PIAAC–L.

PIAAC 2012 assessed skills in literacy, numeracy and problem solving in technology-rich environments. The default option for the skills assessment in PIAAC was a computer-based assessment. After finishing the personal interview, individuals were given a laptop computer and asked to take the skills assessment under the supervision of the interviewer. Respondents with no computer skills or extremely limited computer experience were given a pencil-and-paper version of the skills assessment. There was no time limit on solving the tasks.

For time and cost reasons, each person participating in PIAAC received only a small number of test items in literacy, numeracy or problem solving. Using item response theory (IRT), it was possible to estimate proficiency in skills for each person, even if they did not work on the same items [1]. In order to take measurement error into account, an empirically derived distribution of proficiency values conditional on observed item response patterns and background variables was constructed for each respondent [2]. Subsequently, 10 plausible values, which were multiple imputations, were drawn at random for each respondent from this posteriori distribution. With the data collection in the additional waves of PIAAC-L in 2014 and 2015, additional background variables became available and a new set of plausible values based on the assessment of skills in 2012 and all information from 2012, 2014, and 2015 was generated [3].

For each skill domain, separate proficiency scales were developed using IRT and latent regression models. The results of the assessment are reported separately for each domain on a 0–500 scale. Each scale is constructed with a mean of 250 points and a standard deviation of 50 points. For better comparison, the scales can be divided into five levels in the case of literacy and numeracy and three levels in the case of problem solving.

Additional data on literacy and numeracy were collected in the second wave of PIAAC-L (2015) using instruments from PIAAC 2012. The repeated measurement of PIAAC literacy and

numeracy was included in an extended scaling and analysis model to enable longitudinal analyses with a second set of 10 plausible values [3].

For our analysis, we used all 10 plausible values based on the skills assessment in 2012 and background information from PIAAC 2012 and PIAAC-L 2014 and 2015 as our skills measurement at t_1 and the plausible values based on the skills assessment and questionnaire from PIAAC-L 2015 as our skill measurement at t_2.

References

1. von Davier M, Gonzalez E, Mislevy R. What are plausible values and why are they useful. IERI Monograph Series. 2009;2:9–36.
2. Lüdtke O, Robitzsch A. Eine Einführung in die Plausible-Values-Technik für die psychologische Forschung. Diagnostica. 2017;63(3):193–205. German.
3. Rammstedt B, Martin S, Zabal A, Carstensen C, Schupp J. The PIAAC longitudinal study in Germany: Rationale and design. Large-scale Assessments in Education. 2017;5(1):1–11.
